# Isolation of Human Osteal Macrophages

**DOI:** 10.3390/life16030376

**Published:** 2026-02-27

**Authors:** Juliana Franziska Bousch, Stefanie Lichtenberg, Matthis Schnitker, Jenny Schlösser, Christoph Viktor Suschek, Uwe Maus, Christoph Beyersdorf

**Affiliations:** 1 Department for Orthopedics and Trauma Surgery, Medical Faculty, University Hospital Düsseldorf, Heinrich Heine University Düsseldorf, 40225 Dusseldorf, Germany; 2 Department of Neurology, Medical Faculty, University Hospital Düsseldorf, Heinrich Heine University Düsseldorf, 40225 Düsseldorf, Germany; 3 Core Facility Flow Cytometry, Medical Faculty, University Hospital Düsseldorf, Heinrich Heine University Düsseldorf, 40225 Düsseldorf, Germany

**Keywords:** macrophages, bone metabolism, osteomacs, isolation, inflammation

## Abstract

Osteal macrophages (“osteomacs”) are resident bone macrophages that support osteoblast differentiation and bone formation. Despite their importance in bone homeostasis, their function in human bone metabolism and osteoporosis remains poorly understood, largely due to the lack of a standardized isolation protocol. Here, we present a protocol for isolating primary human osteomacs from femoral head specimens obtained during arthroplasty. After the removal of bone marrow to minimize contamination with marrow-derived macrophages, bone fragments were enzymatically digested and osteomacs were isolated using CD14-based MACS^®^ or CD14/CD45/ALP-based FACS. Immunofluorescence confirmed macrophage identity and revealed expression of markers associated with both M1-like and M2-like activation states. Isolated cells displayed heterogeneous morphology and could be maintained in culture. This protocol enables reproducible isolation of human osteomacs and provides a foundation for translational studies investigating osteoimmune interactions in bone disease and osteoporosis.

## 1. Introduction

Osteoporosis is the most common metabolic bone disease worldwide and is associated with significant morbidity. Since the introduction of the term “osteoimmunology” in 2000, the interaction between inflammatory processes and bone metabolism has increasingly become the focus of research [1]. Despite substantial progress in understanding these interactions, effective therapeutic approaches to counteract inflammation-induced bone loss remain limited.

Macrophages play a central role in linking the immune system and bone metabolism. Several years ago, a population of tissue-resident macrophages in bone was described [2]. These osteal macrophages, commonly referred to as “osteomacs”, are critically involved in bone metabolism. Located in close proximity to mature osteoblasts at sites of active bone remodeling, they form a characteristic “canopy structure” around osteoblasts during bone formation. Osteomacs (OMs) are essential for the differentiation and mineralization of osteoblasts, as demonstrated both in vitro and in vivo [2]. However, their role in the pathophysiology of osteoporosis, particularly in inflammation-induced bone loss, remains largely unexplored.

A hallmark feature of macrophages is their remarkable plasticity. Depending on local environmental cues, macrophages can adopt pro-inflammatory (classically termed M1-like) or anti-inflammatory and tissue-supportive (M2-like) activation states, although macrophage polarization is now increasingly understood as a continuum rather than a binary classification [3,4]. Their function in bone metabolism is dependent on polarization: while M2-like polarization promotes bone formation, pro-inflammatory M1-like polarization favors bone resorption [5]. In this context, osteomac polarization may represent both a sensitive biomarker of the inflammatory bone microenvironment and a potential therapeutic target to modulate bone remodeling in osteoporosis. However, which osteomac subtypes are present in human osteoporotic bone and how their functional states can be experimentally or therapeutically manipulated remain largely unknown.

While recent research has established a protocol for isolating murine osteomacs using the mouse-specific macrophage marker F4/80 [6], no standardized method exists for the isolation of human osteomacs to date. In this study, we present a novel isolation method for human osteomacs, adapted from existing protocols for primary osteoblast isolation. Previous studies have demonstrated that murine osteomacs are co-isolated during osteoblast extraction, constituting approximately 15.9% of the cell population [2]. Establishing a reliable method for isolating human osteomacs is essential to advance our understanding of their role in osteoporosis and develop targeted therapeutic interventions.

## 2. Experimental Design

### 2.1. Isolation Procedure of Primary Human Osteomac

Bone samples were obtained from femoral heads of patients undergoing endoprosthetic treatment due to coxarthrosis or femoral neck fracture. To minimize contamination by bone marrow-derived macrophages (BMDMs), the bone marrow was thoroughly removed by repeated washing. Osteomacs seem to be distributed along endosteal and periosteal bone surfaces [2]. Therefore, this washing step was crucial to ensure the isolation of resident bone macrophages while minimizing interference from circulating or marrow-derived cells.

Following bone marrow removal, an enzymatic digestion was performed using a combination of collagenase II and dispase to release cells embedded in the bone matrix. The bone fragments were incubated in the enzyme solution, and every 15 min, the supernatant was collected and centrifuged to harvest the released cells. Fresh collagenase solution was added to the remaining bone fragments for continued digestion. This process was repeated for a total of five fractions, while the first fraction, containing surface contaminants and residual marrow components, was discarded to improve the purity of the final cell populations.

**Figure 1:**
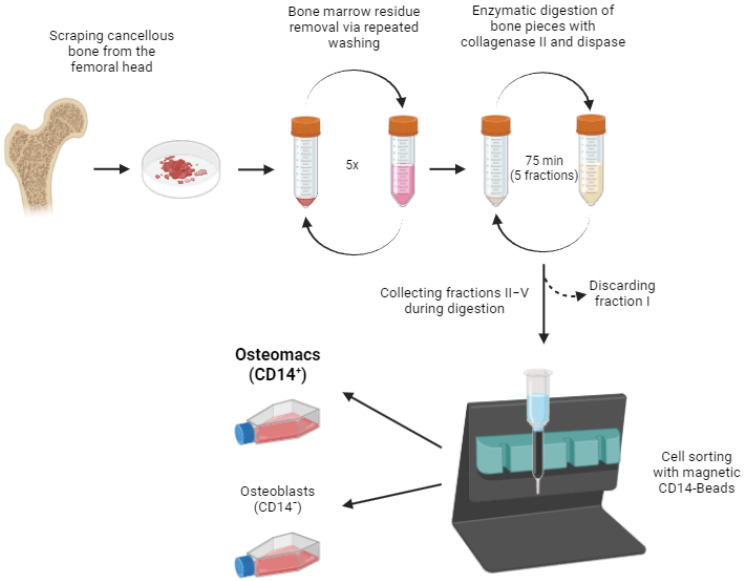
Schematic representation of the study protocol. The detailed protocol is described in the methods section (Step-by-Step protocol for the isolation of osteomacs). Image created in BioRender. Bousch, J. (2025) https://BioRender.com/u93k231 (accessed on 10 July 2025).

To separate osteoblasts from osteomacs, magnetic-activated cell sorting (MACS^®^) was employed. Cells from the pooled fractions II-V were labeled with CD14-conjugated magnetic beads, allowing for specific separation based on the surface expression of CD14. The resulting cell fractions comprised a CD14-negative population (osteoblasts) and a CD14-positive population (osteomacs). This strategy resulted in a strong enrichment of both cell types, reducing cross-contamination (Figure 1). A step-by-step protocol is provided in Section 3. After separation, the isolated cell populations were cultured under standard conditions to allow cell attachment and recovery.

However, the MACS-based isolation procedure led to contamination of the osteomac cultures with osteoblasts, which had a higher proliferative capacity and overgrew the macrophage population after some time. Therefore, it is recommended to use the isolated osteomacs immediately after MACS for experiments without further passaging.

**Figure 2:**
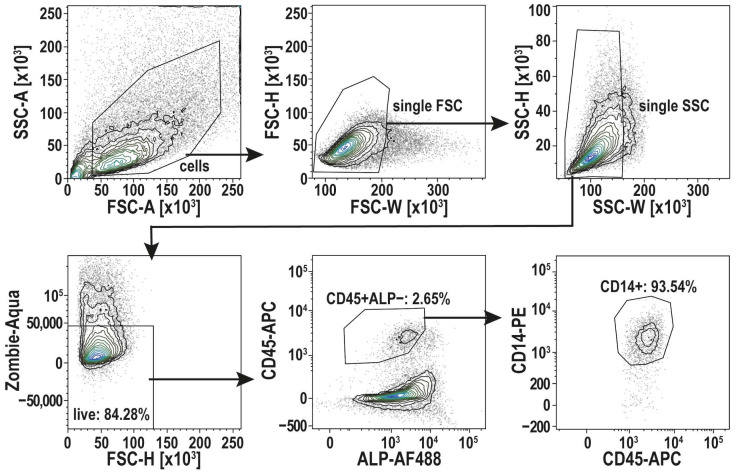
Gating strategy for cell sorting of osteomacs (CD45^+^ ALP^−^ CD14^+^). After Gating of single cells, using the width of the forward and side-scatter signal (FSC-W, SSC-W), living cells were selected (Zombie-Aqua-). CD45^+^ ALP^−^ cells were then selected to identify the osteomac population by CD14^+^. Those cells were selected for sorting. Shown here is a representative FACS run. This FACS protocol was established with *n* = 3 donors.

Alternatively, to address this issue, we performed fluorescence-activated cell sorting (FACS) to ensure a highly enriched macrophage population for analyzing the proliferation capacity of osteomacs without osteoblast contamination. After the isolation of osteoblasts and osteomacs, as described in steps 1–8 of the “Step-by-Step Protocol for the Isolation of osteomacs.”, the cells were cultivated until confluency. These confluent cultures were detached with Accutase and filtered through a 40 µm cell strainer. After blocking Fc receptors to avoid non-specific antibody binding, the cells were stained with a Live/Dead staining and fluorophore-conjugated antibodies for CD14 (Clone REA599), CD45 (Clone 2D1), and ALP (Clone 8B6). Using a BD FACSymphonyTM S6, the osteomac cell fraction (CD14^+^ CD45^+^ ALP^−^) was sorted in α-MEM with 1% Pen/Strep and 20% FCS supplemented with 10 ng/mL M-CSF. ALP was stained on live cells as a surface marker (no fixation/permeabilization) and was used exclusively to exclude ALP^+^ osteoblasts during gating of the CD45^+^CD14^+^ population. Single-stained controls were used for compensation. The gating strategy for cell sorting is depicted in Figure 2.

### 2.2. Characterization of Isolated Primary Human Osteomacs

**Figure 3:**
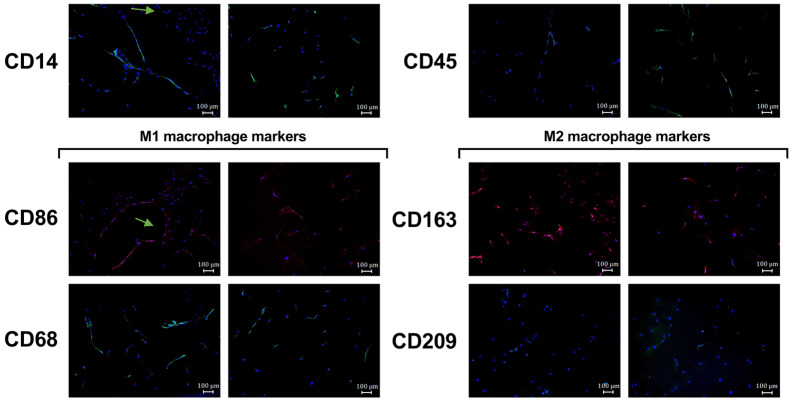
Macrophage surface marker expression of osteomacs isolated by MACS^®^. The immunofluorescence (IF) images show staining for the macrophage markers CD14, CD45 and markers for M1-like (CD86 and CD68) and M2-like macrophage polarization (CD163, CD209) together with nuclei staining by using DAPI (blue). Arrows indicate thread-like configuration of some osteomacs. Shown here are representative IF images. IF staining was performed with *n* = 2 donors.

To confirm the phenotype of the isolated osteomac culture, several macrophage surface markers were analyzed by immunofluorescence staining. CD14 and CD45 were used as general hematopoietic and monocyte/macrophage markers serving as controls. CD68 and CD86 were assessed as markers for M1-like macrophages, while CD163 and CD209 were used to identify M2-like macrophage activation. Both M1 and M2 surface markers could be identified in the isolated osteomac cultures (Figure 3). Interestingly, it was observed that the osteomacs occasionally arranged themselves in close proximity to one another, forming a thread-like configuration.

**Figure 4:**
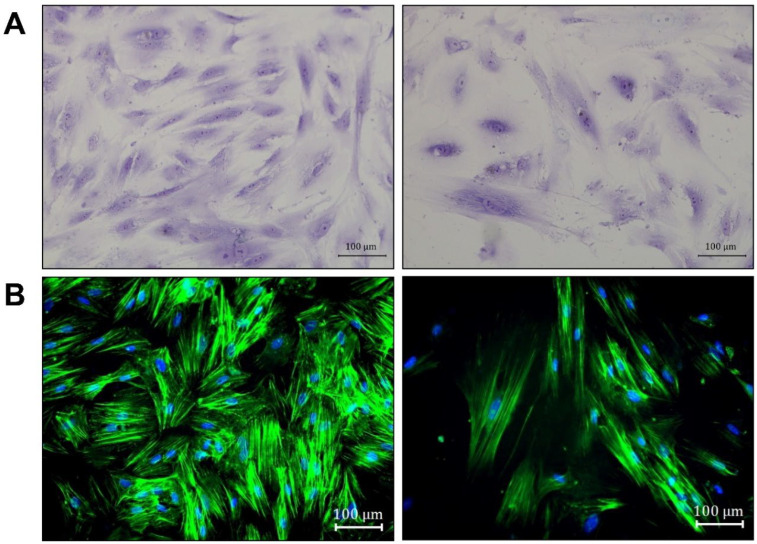
Morphological features of osteomacs isolated by FACS. The cells were stained with (**A**) toluidine blue and (**B**) the f-actin of the cells was stained with phalloidin (green) and the nuclei by using DAPI (blue). The images were taken with a bright-field microscope at 200× magnification (scale bar = 100 µm). Arrows indicate heterogeneous morphology (orange: thin; green: flattened shape). Toluidine and phalloidine staining was representatively performed with a single donor.

Cells sorted by FACS were used to characterize the morphological features of osteomacs in culture. Toluidine blue staining of these cells revealed heterogeneous morphologies, with some cells appearing thin and spread, while others exhibited a flattened shape (Figure 4A). Notably, osteomacs displayed prominent stress fibers composed of dense and elongated actin filaments (Figure 4B).

## 3. Materials and Equipment

### 3.1. Materials

The cell culture materials were obtained from Sarstedt AG & Co. KG (Nümbrecht, Germany). Unless otherwise specified, all other materials and reagents were obtained from Merck KGaA (Darmstadt, Germany).

### 3.2. Bone Material, Ethics Approval, and Patient Information

The isolation and use of human osteomacs was approved by the local Research Ethics Committee of the Heinrich Heine University Düsseldorf (Study No. 5585R). The patients had given written consent and had undergone arthroplasty due to osteoarthritis or fracture at the Clinic for Orthopedic and Trauma Surgery at the University Hospital of Düsseldorf (Germany). Femoral heads from patients acquired during surgery were stored in phosphate-buffered saline (PBS) with 1% penicillin/streptomycin (Pen/Strep) by PAN-Biotech GmbH (Aidenbach, Germany) at 4 °C until isolation. The isolation was performed on the day following the surgery, as extended waiting could have reduced cell number and purity.

### 3.3. Step-by-Step Protocol for the Isolation of Osteomacs

Material:

Wash Medium: Dulbecco’s Modified Eagle Medium (DMEM), high-glucose without phenol red (Gibco^®^ by Life TechnologiesTM, Carlsbad, CA, USA) with 1% Penicillin/Streptomycin (Pen/Strep)
Wash Buffer: phosphate-buffered solution (PBS) with 1% Pen/Strep and 1% fetal bovine serum (FBS) by PAN-Biotech GmbH (Aidenbach, Germany)
Collagenase II (2.5 mg/mL) and Dispase (2.5 mg/mL) in Wash Medium
MACS buffer: PBS with 0.5% Bovine Serum Albumine (BSA) and 2 mM EDTA (pH = 7.2)
OM Medium: alpha-Minimum Essential Medium Eagle (α-MEM) with 10% FBS, 1% Pen/Strep and 10 ng/mL macrophage-stimulating factor (M-CSF)
OB Medium: DMEM with 10% FBS, 1% Pen/Strep, 1% HEPES

Procedure:

(1) The cancellous bone from human femoral heads was scraped out and the bone pieces were transferred into a 50 mL Falcon tube. In a representative isolation, the retrieved tissue mass was approximately 5 mg, but may vary between donors due to differences in tissue firmness and ease of retrieval. Supplementary Tables S1–S3 report the cell yields and donor characteristics for each isolation.

*Bone marrow removal* (for optional use for bone marrow-derived macrophage (BMDM) or bone morrow-derived mesenchymal stem cells (BMSC) isolation):

(2) The bone pieces were rinsed with 25 mL Wash Buffer and vigorously shaken. The supernatant was discarded.
(3) 20 mL of Wash Medium was added and the falcon was placed on a tube roller for 10 min at 37 °C. The supernatant was discarded.
(4) 20 mL of Wash Medium was added and rotated again for 10 min (37 °C). The supernatant was discarded.
(5) Steps 3 and 4 were repeated.

*Release of osteoblasts and osteomacs from bone matrix by collagenase digestion:*

(6) 8 mL Collagenase II/Dispase solution was added to the bone pieces and incubated on a tube roller for 15 min at 37 °C. The supernatant (fraction I) was discarded.
(7) Step 6 was repeated four times, each time using a fresh Collagenase II/Dispase solution. After digestion, the supernatant of each fraction was centrifuged at 400× *g* for 5 min to remove the enzymes. The cell pellet was resuspended in OB Medium and the cells from fractions II–V were pooled.
(8) The pooled cell suspension was filtered through a 70 µm cell strainer.

*Isolation of Osteomacs by MACS^®^, based on the protocol by Miltenyi Biotec* (Bergisch Gladbach, Germany):

(9) Cells were centrifuged at 400× *g* for 5 min and transferred to a 2 mL reaction tube.
(10) Cells were centrifuged again and resuspended in 80–160 µL MACS-Puffer (PBS with 0.5% BSA and 2 mM EDTA, pH = 7.2) depending on the cell number. 20–40 µL CD14-Beads (Miltenyi Biotec) were added and incubated for 15 min at 4 °C.
(11) An MS column was prepared by placing it into the magnetic field of a MiniMACSTM Separator and rinsing it with 500 µL MACS buffer.
(12) The cell suspension was carefully applied into the column and washed three times with 500 µL MACS buffer. CD14-negative osteoblasts were eluted during the washing steps.
(13) The column was removed from the magnet and the CD14-positive Osteomacs were eluted by rinsing with 1 mL of MACS buffer.
(14) After centrifugation (400× *g*, 5 min), the osteomacs were resuspended in OM Medium and seeded into a T75 cell culture flask. The OM-depleted osteoblast culture was resuspended in OB Medium and seeded into a T175 cell culture flask. The cell cultures were maintained in a humidified chamber at 37 °C with 5% CO_2_ with medium changes twice per week.

### 3.4. Fluorescence-Activated Cell Sorting

After centrifugation at 400× *g* for five minutes, the cells were resuspended in 100 µL of Fc Block^TM^ (BD Biosciences, Franklin Lakes, NJ, USA) and incubated for ten minutes. After another centrifugation, the cells were incubated with Zombie Aqua (BioLegend, San Diego, CA, USA) for ten minutes for cell viability staining. The cells were incubated with the fluorophore-conjugated antibodies CD14 (Clone REA599), CD45 (Clone 2D1), and ALP (Clone 8B6) (see Table 1). The total volume was brought up to 75 µL with FCS and the cells were incubated on ice for 30 min. To wash the cells, 4 mL of sorting buffer (PBS with 0.5% BSA and 2 mM EDTA) was added. After centrifugation, the cell pellet was resuspended in 300 µL of sorting buffer for FACS cell sorting. Cells were filtered through a 100 µm cell strainer before sorting. Cell sorting was performed on a BD FACSymphony™ S6 (6-laser configuration: 355 nm, 405 nm, 445 nm (deactivated), 488 nm, 561 nm, 633 nm; Software FACS DIVA v.9.5.1) with the 100 µm nozzle at an event rate of 2000 evts/sec. The osteomac cell fraction (CD14^+^ CD45^+^ ALP^−^) was sorted into a 96-well plate (2200 cells per well, purity mode) in α-MEM with 1% Pen/Strep and 20% FCS supplemented with 10 ng/mL M-CSF.

### 3.5. Immunfluorescence Staining

Osteomacs were seeded at a density of 1 × 10^4^ cells per well on glass coverslips in a 24-well cell culture plate and incubated for 48 h. Cells were subsequently fixed with 4% paraformaldehyde (PFA) for 10 min at room temperature. Blocking and permeabilization was performed for 1 h at 37 °C in PBS containing 0.1% Triton X-100 and 5% normal goat serum (NGS). Primary antibodies targeting CD14, CD45, CD68, CD86, CD163, and CD209 were diluted in blocking solution and incubated overnight at 4 °C. Information about used antibodies and dilutions are listed in Table 2. After three washes with PBS, fluorophore-conjugated secondary antibodies (goat anti-mouse or goat anti-rabbit; 1:200 in PBS) were applied for 1 h at room temperature. Following another three PBS washes, coverslips were mounted on glass slides using Mowiol 4-88 containing DAPI (1:1000). Fluorescence images were taken using a Keyence BZ-X800 fluorescence microscope equipped with a 10× objective.

### 3.6. Toluidine and Phalloidin Staining

OMs were seeded at 50% confluency in appropriate culture vessels. Cells were fixed with 4% paraformaldehyde (PFA) for 15 min at room temperature (RT) and washed with phosphate-buffered saline (PBS). For toluidine blue staining, cells were incubated with 0.3% toluidine blue solution for 1–3 min at RT. Excess dye was removed by rinsing the cells with double-distilled water (ddH_2_O) until the wash solution appeared clear. For actin filament visualization, cells were permeabilized with 0.1% Triton-X-100 for 10 min at RT. Cells were then incubated with Alexa Fluor Plus 647-phalloidin (Invitrogen, Carlsbad, CA, USA) for 60 min at RT. Following incubation, cells were counterstained with DAPI (1:1000) for 10 min at RT and washed with PBS. Fluorescence and bright-field images were taken using a Keyence BZ-X800 microscope (Keyence, Osaka, Japan) using a 20× objective.

## 4. Expected Results

The isolation method for human osteomacs described here facilitates detailed investigation of a cell population that, despite its central role in bone homeostasis, remains insufficiently characterized in humans. By adapting and refining existing osteoblast isolation protocols, our approach allows for the reliable extraction of Osteomacs from human bone tissue, providing a critical tool for translational research in osteoimmunology. Given the high plasticity of macrophages and their ability to respond dynamically to local microenvironmental cues, the method enables the study of interindividual differences in Osteomac phenotype and function.

However, isolating osteomacs from an osteoblast co-culture comes with several limitations. The MACS sorting did not yield completely pure cultures and still showed contamination with osteoblasts to some extent. The main challenge is that osteoblasts proliferate more efficiently in vitro than macrophages, so even a small number of incorrectly sorted osteoblasts could rapidly overgrow the culture. Because of the narrow gating parameters required for FACS and the overall low abundance of osteomacs, the final cell yield after FACS is relatively low. Consequently, follow-up experiments are only possible to a limited extent or require prolonged expansion of the isolated cells, an approach that may arguably alter their phenotype and marker expression. The FACS strategy included established macrophage-associated markers (CD14 and CD45), which were used to define the osteomac population prior to imaging. However, immunocytochemical validation of FACS-isolated osteomacs using canonical macrophage markers could not be performed due to the limited number of cells obtained after sorting. Future studies with larger cell yields will be required to allow additional phenotypic validation. Moreover, future studies should also focus on systematically quantifying cell composition and purity at multiple stages of isolation and culture, including longitudinal assessment of CD14^+^CD45^+^ALP^−^ cells and potential osteoblast contamination. Such benchmarking will require larger input cell numbers and extended multicolor flow cytometric analyses.

With respect to method selection, MACS and FACS address complementary experimental needs. MACS is particularly well suited for rapid macrophage enrichment when high cell yield, viability, and minimal processing time are prioritized, for example in short-term functional assays performed immediately after isolation. In contrast, FACS is preferable for applications requiring high purity and precise phenotypic resolution, such as purity-sensitive downstream analyses including transcriptomic or epigenetic profiling. Moreover, FACS allows the exclusion of dead cells and undesired cell populations based on multiple markers, albeit at the cost of longer processing times and potentially increased cellular stress.

For cell isolation, we relied on the applied isolation protocol in combination with the pan-macrophage markers CD14 and CD45, as no specific markers for human osteomacs have been identified to date. While we consider this approach to be appropriate and feasible, it must be acknowledged that potential contamination with other CD14/CD45-positive cell populations, such as bone marrow–derived macrophages or hematopoietic precursor cells, cannot be entirely excluded. Beyond the morphological characterization presented here and basic proliferative and phenotypic characterizations (Figures S1 and S2), as well as the absence of osteoclastogenesis in cultures treated with M-CSF and RANKL (Figure S3), future studies should therefore include in-depth transcriptomic profiling to more comprehensively define these cells and to identify potentially specific markers for human osteomacs.

The immunofluorescence analyses revealed expression of markers commonly associated with both M1-like (CD68, CD86) and M2-like (CD163, CD209) macrophage activation states in the isolated osteomac population. However, the present data do not allow discrimination between co-expression of these markers within individual cells and the presence of distinct macrophage subpopulations. Therefore, the observed marker patterns should be interpreted as a qualitative description of phenotypic diversity rather than as definitive evidence of cellular heterogeneity. Future studies employing quantitative single-cell–based approaches, including systematic marker co-expression analysis and spatial profiling across multiple donors and defined bone regions, will be required to more precisely characterize osteomac heterogeneity and functional states in human bone.

We consistently observed an increase in cell number and sustained survival in culture of human osteomacs (Figure S1), as has also been described for other macrophage subsets under certain conditions [7]. Osteomacs proliferated spontaneously without M-CSF, with no significant difference compared to M-CSF–treated cultures (Figure S5). However, long-term phenotypic stability under M-CSF–free conditions has not been established. The osteomacs morphology in culture appeared heterogeneous, showing flattened cell shapes and long filopodia. Other macrophage subtypes are known to vary morphologically depending on their polarization state. For example, Pelegrin and Surprenant described a morphology similar to that observed here following M1 polarization of peritoneal macrophages, while Vereyken et al. reported a stretched and elongated phenotype in M2-polarized BMDMs [8,9]. Using machine learning approaches, it is now possible to distinguish macrophage subtypes solely based on morphological characteristics [10]. These approaches may be applied in future studies to rapidly and efficiently identify osteomac phenotypes. However, it remains to be determined how osteomac morphology varies in response to environmental cues and polarization states.

Phenotypic differences can also be reflected in the organization of the actin cytoskeleton. A biphasic contraction of actin–myosin fibers has been reported upon inflammatory activation. In activated macrophages, the actin cytoskeleton is typically concentrated around the nucleus, whereas in non-activated cells it appears more evenly distributed throughout the cell, such as in the osteomacs observed here [11].

Human macrophages frequently co-express both M1 and M2 markers [12,13] and, unlike their murine counterparts, are often distinguishable only by quantitative differences in expression. Consistent with this, we also observed expression of both marker types in osteomacs. Thus, combining morphological analysis with marker profiling could provide valuable complementary information for distinguishing different osteomac phenotypes.

The availability of human osteomacs for in vitro experiments may allow investigation of how patient-related factors, such as age, comorbidities, or systemic inflammation, influence the osteoimmune interface. In the long term, we expect that the integration of osteomac-specific phenotype profiles with clinical metadata will allow the identification of disease-specific macrophage signatures. Such insights could lay the groundwork for novel diagnostic biomarkers or therapeutic strategies targeting macrophage subtypes involved in bone loss disorders, including osteoporosis.

## Figures and Tables

**Table 1:** List of fluorophore-labeled antibodies for FACS.

Antibody/Conjugate	Reference Number	Company	Dilution
CD14-PE	130-110-519	Miltenyi Biotec	1/50
CD45-APC	340910	Miltenyi Biotec	1/15
ALP-Alexa Fluor 488	NB110-3638AF488	Novus Biologicals (Littleton, CO, USA)	1/15

**Table 2:** List of primary antibodies for immunofluorescence staining.

Antibody (Clone)	Host/Target Species	Reference Number	Company	Dilution
CD14 (1H5D8)	mouse anti-human	ABIN1724922	Antibodies-online GmbH (Aachen, Germany)	1/500
CD45 (2B11)	mouse anti-human	sc-20056	Santa Cruz Biotechnology (Dallas, TX, USA)	1/50
CD68 (KP1)	mouse anti-human	sc-20060	Santa Cruz Biotechnology	1/50
CD209 (UW60.1)	mouse anti-human	AM26379PU-N	OriGene Technologies, Inc. (Rockville, MD, USA)	1/50
CD86	rabbit anti-human	bs-1035R	Bioss Antibodies (Woburn, MA, USA)	1/100
CD163	rabbit anti-human	bs-23127R	Bioss Antibodies	1/100

## Data Availability

The raw data supporting the conclusions of the article will be made available by the authors on request.

## Supplementary Materials

The following supporting information can be downloaded at: https://www.mdpi.com/article/10.3390/life16030376/s1, Figure S1: Proliferation capacity of FACS-sorted osteomacs in passage 6 (A) and their reduction of viability from passage 4 to 6 (B), measured by MTT assays (n = 1); Figure S2: Enhanced TNF-α concentration in the supernatant of FACS-sorted osteomacs treated with LPS (100 ng/mL) for 2 or 24 h, quantified by ELISA (n = 1); Figure S3: TRAP staining of FACS-sorted osteomacs after incubation with RANKL (50 ng/mL) and M-CSF (25 ng/mL) for 21 days; Figure S4: Comparison of M1-type and M2-type marker expression in MACS-sorted osteomacs, quantified by qPCR, normalized on GAPDH expression (n = 3–4); Figure S5: Proliferation capacity of FACS-sorted osteomacs with and without M-CSF addition (10 ng/mL), measured by MTT assay (n = 1); Table S1: Cell yield list of MACS-Sortings; Table S2: Cell yield list of FACS-Sortings; Table S3: Characteristics of donors used for MACS and FACS-Sorting.

## Abbreviations

The following abbreviations are used in this manuscript:

α-MEM	alpha-Minimum Essential Medium Eagle
BMDM	Bone Marrow Derived Macrophages
BSA	Bovine Serum Albumine
FACS	Fluorescence Activated Cell Sorting
M-CSF	Macrophage Stimulating Factor
MACS	Magnetic Activated Cell Sorting
NGS	Normal Goat Serum
OM	Osteomacs
PFA	Paraformaldehyde
Pen/Strep	1% Penicillin/Streptomycin
PBS	Phosphate-Buffered Saline
