# Supplementary material for: Isolation of Human Osteal Macrophages
Source: Life (Basel). 2026 Feb 27;16(3):376. doi: 10.3390/life16030376 (PMC13027567; doi:10.3390/life16030376)
Supplement: Supplementary file 1 [file life-16-00376-s001.zip › life-4160576-supplementary.pdf]

Supplements

List of Figures:

Figure S1: Proliferation capacity of FACS-sorted osteomacs in passage 6 (A) and their reduction of viability from passage 4 to 6 (B), measured by MTT assays (n = 1).

Figure S2: Enhanced TNF-α concentration in the supernatant of FACS-sorted osteomacs treated with LPS (100 ng/mL) for 2 or 24 hours, quantified by ELISA (n = 1).

Figure S3: TRAP staining of FACS-sorted osteomacs after incubation with RANKL (50 ng/mL) and M-CSF (25 ng/mL) for 21 days.

Figure S4: Comparison of M1-type and M2-type marker expression in MACS-sorted osteomacs, quantified by qPCR, normalized on GAPDH expression (n = 3-4).

Figure S5: Proliferation capacity of FACS-sorted osteomacs with and without M-CSF addition (10 ng/mL), measured by MTT assay (n = 1).

List of Tables:

Table S1: Cell yield list of MACS-Sortings.

Table S2: Cell yield list of FACS-Sortings.

Table S3: Characteristics of donors used for MACS and FACS-Sorting.

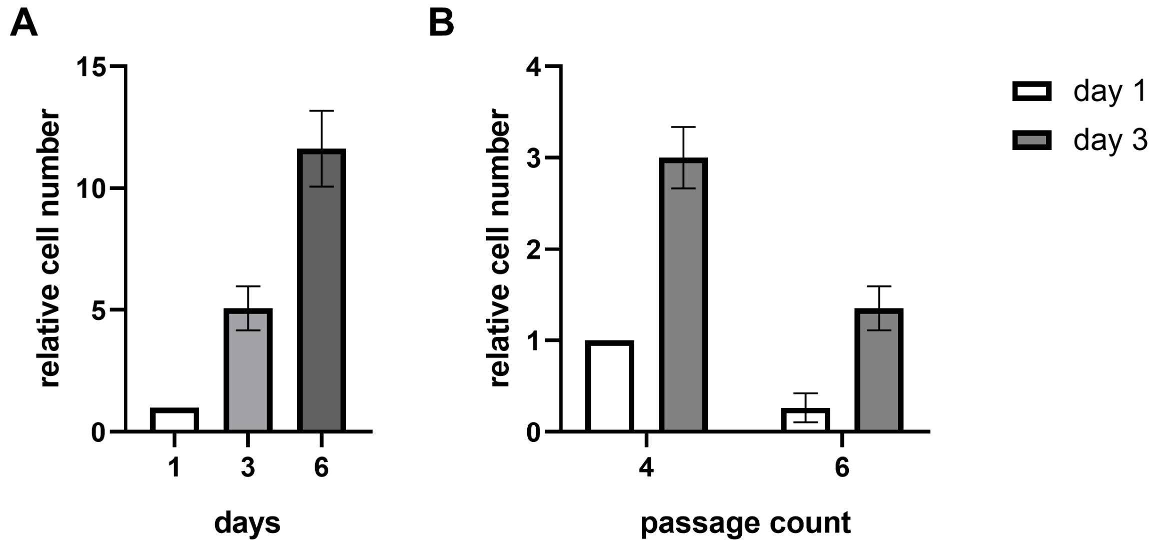

Figure S1: Proliferation capacity of FACS-sorted osteomacs in passage 6 (A) and their reduction of viability from passage 4 to 6 (B), measured by MTT assays (n = 1).

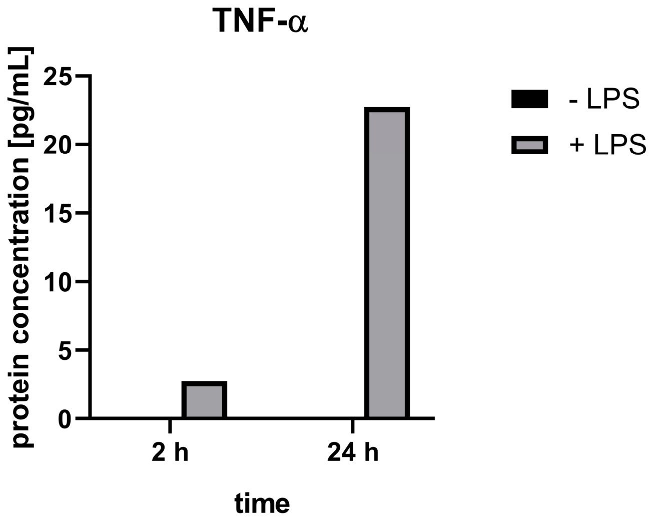

Figure S2: Enhanced TNF-α concentration in the supernatant of FACS-sorted osteomacs treated with LPS (100 ng/mL) for 2 or 24 hours, quantified by ELISA (n = 1).

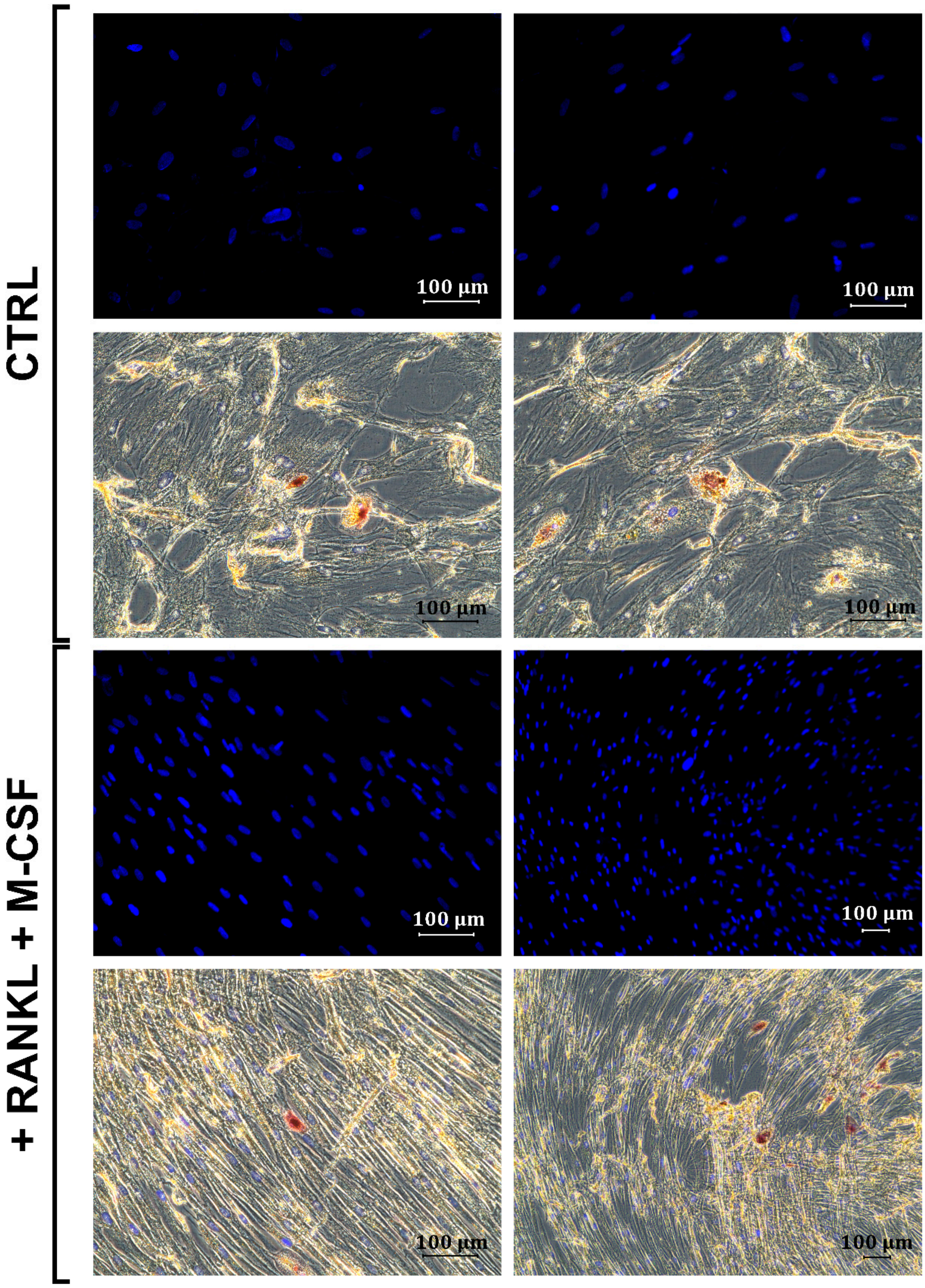

Figure S3: TRAP staining of FACS-sorted osteomacs after incubation with RANKL (50 ng/mL) and M-CSF (25 ng/mL) for 21 days.

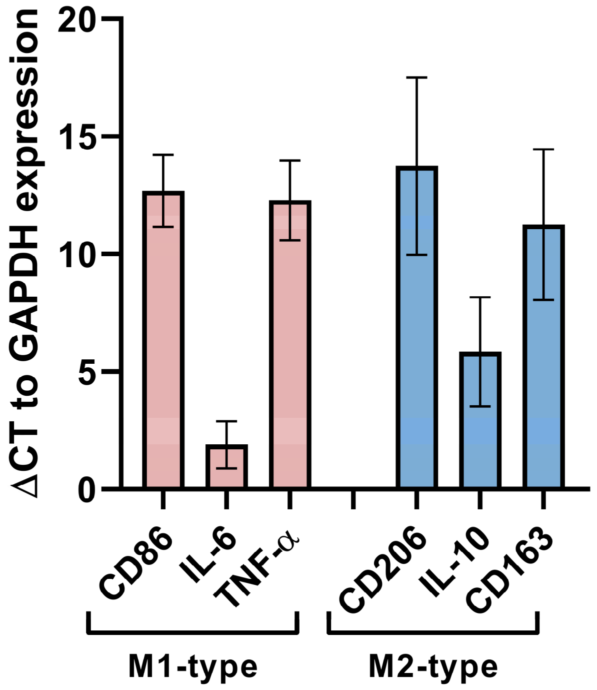

Figure S4: Comparison of M1-type and M2-type marker expression in MACS-sorted osteomacs, quantified by qPCR, normalized on GAPDH expression (n = 3-4).

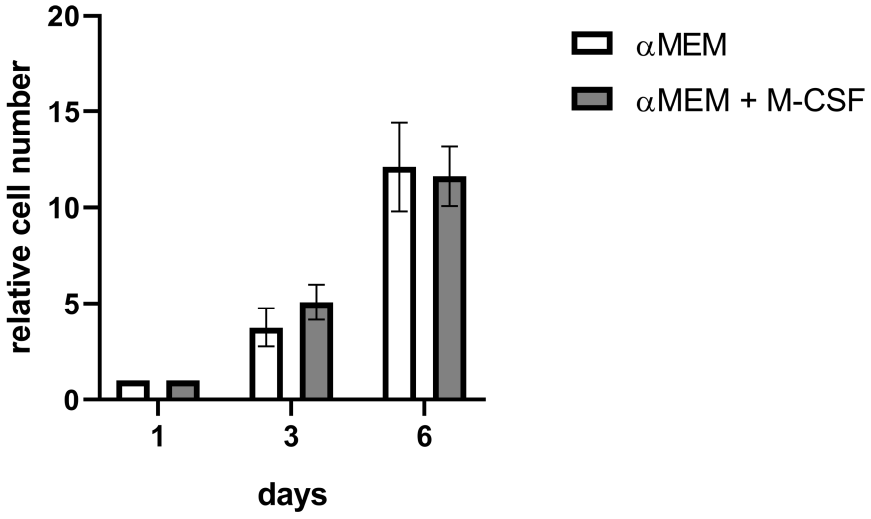

Figure S5: Proliferation capacity of FACS-sorted osteomacs with and without M-CSF addition (10 ng/mL), measured by MTT assay (n = 1).

Table S1: Cell yield list of MACS-Sortings.

Donor	*Total cells pre-sorting [n]*	*$CD14^+$ cells [n]*	*$CD14^+$ cells [%]*
OM_MACS_1	7.3×10^7	2.2×10^5	2.68
OM_MACS_2	5.4×10^7	1.1×10^5	2.41

Table S2: Cell yield list of FACS-Sortings.

Donor	*Isolation to sorting [days]*	*Total cells pre-sorting [n]*	*$CD14^+CD45^+ALP^-$ cells [n]*	*$CD14^+CD45^+ALP^-$ cells [%]*
OM_FACS_1	16	3×10^6	3×10^3	0.1
OM_FACS_2	20	2.25×10^6	6.5×10^3	0.29
OM_FACS_3	27	3.55×10^6	37×10^3	1.04

Table S3: Characteristics of donors used for MACS and FACS-Sorting.

Donor	*Age*	*Sex*	*Diagnosis*	*Medication*
OM_MACS_1	87	f	fracture	Omeprazole, Captopril
OM_MACS_2	72	m	osteoarthritis	Sitagliptin, Metformin, Metoprolol, Ramipril, Amlodipine, ASA, Allopurinol, Simvastatin
OM_FACS_1	72	m	osteoarthritis	Atorvastatin, ASA, Ramipril
OM_FACS_2	66	m	osteoarthritis	Tamsulosin
OM_FACS_3	54	f	osteoarthritis	Clopidogrel, VitD, Iron, Folic acid, Lorazepam, Ramipril, Tilidine, Indacaterol/Glycopyrronium
